# Developing A Case-Based Reasoning Model for Safety Accident Pre-Control and Decision Making in the Construction Industry

**DOI:** 10.3390/ijerph16091511

**Published:** 2019-04-29

**Authors:** Yikun Su, Shijing Yang, Kangning Liu, Kaicheng Hua, Qi Yao

**Affiliations:** 1 School of Civil Engineering, Northeast Forestry University, Harbin 150040, China; suyikun@nefu.edu.cn (Y.S.); yaoqi103069@163.com (Q.Y.); 2 School of Civil Engineering, Harbin Institute of Technology, Harbin 150001, China; corninghit@163.com; 3 Guangdong Huiqing Expressway Co., Ltd., Guangzhou 510900, China; 13363236025@163.com

**Keywords:** case-based reasoning (CBR), construction safety management, hazard prevention, pre-control and decision making

## Abstract

Case-based reasoning (CBR) has been extensively employed in various construction management areas, involving construction cost prediction, duration estimation, risk management, tendering, bidding and procurement. However, there has been a dearth of research integrating CBR with construction safety management for preventing safety accidents. This paper proposes a CBR model which focuses on case retrieval and reuse to provide safety solutions for new problems. It begins with the identification of case problem attribute and solution attribute, the state of hazard is used to describe the problem attribute based on principles of people’s unsafe behavior and objective’s unsafe state. Frame-based knowledge representation method is adopted to establish the case database from dimensions of slot, facet and facet’s value. Besides, cloud graph method is introduced to determine the attribute weight through analyzing the numerical characteristics of expectation value, entropy value and hyper entropy value. Next, thesaurus method is employed to calculate the similarity between cases including word level similarity and sentence level similarity. Principles and procedures have been provided on case revise and case retain. Finally, a real-world case is conducted to illustrate the applicability and effectiveness of the proposed model. Considering the high potential for pre-control and decision-making of construction safety accident, the proposed model is expected to contribute safety managers to take decisions on prevention measures more efficiently.

## 1. Introduction

The construction industry has a very poor reputation due to its high safety risks [1,2]. According to the accident reports published by the Ministry of Housing and Urban-Rural Development (http://ginfo.mohurd.gov.cn/), 2355 construction accidents occurred in China from 2012 to 2016, causing a total of 3168 deaths [1,3]. Prior studies have reached a consensus that fall accidents are the predominant type of construction accident, accounting for roughly 50 percent of all types of accidents [2,4]. The current situation of construction safety management offers a depressing picture, therefore, eliminating construction safety risks should be constantly given priority.

Safety hazards—potential location, situation, equipment or behavior that threaten individuals’ safety—usually have injuries or immediate fatalities as the consequence of incidents. The hazard categories are summarized, including worker behaviors, work environment, materials, equipment, immediate supervision, project management and so on [4,5,6,7]. Safety operation and management are both a challenge or opportunity for construction enterprises. Doing well in safety management can not only avoid the direct losses of accidents, reduce construction risks and improve internal control of enterprises, but also enhance the corporate image and social benefits, promoting the competitiveness and long-term development of enterprises. No matter whether from the perspective of development of enterprises or social performance, it is imperative to strengthen the safety management and control.

Pre-control of safety hazard means recognizing, forecasting and controlling underlying hazards in advance to ensure the hazard is controlled, which is the primary principle of implementing safety management. Hazard prevention strategies should be developed for reducing safety risks in the early stages of process design rather than eliminating hazards after they are detected [8]. Recently, the concept of accident prevention through design, which is look-ahead thinking to identify embedded safety hazards during the design phase, has been paid more attention [9]. How to carry out prevention and control measures of safety accidents is challenging. Generally, hazard prevention still focuses on a traditional way of risk management, ignoring the complicated relationships between accident causation and consequence, depending on a safety manager’s or supervisor’s decision making, and lacking an effective approach for automatically predicting safety accidents. However, the inherent characteristics—objectivity, potentiality, and complexity—of safety hazards makes them difficult to prevent and control just based on people’s knowledge, experience and impulsive decisions. An identical hazard may cause distinct consequences in different times, places and situations. It is imperative to develop an effective information database of safety accidents with the help of IT technology, based on system safety theory.

The Case-Based Reasoning (CBR) technique, a new paradigm of artificial intelligence, has been suggested as a viable method to solve current problems by referring to knowledge, information and experience accumulated from previous similar occasions [10]. It has been demonstrated as an applicable and effective approach in construction management, including construction cost prediction, duration estimation, risk management, tendering, bidding and procurement [11,12,13]. Despite its strong potential as a decision-supporting tool in construction, only a few studies have considered CBR for safety hazard identification and prevention. Based on the literature review in Section 2.1, it can be seen that the CBR technique has potential to improve the efficiency and quality of safety management. Nevertheless, there are still some research gaps need to be filled. First, most CBR models are proposed based on specific scenarios, such as subway projects, the marine industry, etc. The universality of these models needs to be further studied. Additionally, few studies establish a CBR-based model of construction safety management from the perspective of hazard prevention. Second, the innovative methods concerning weight determination of case attributes and similarity calculations are deficient. Third, prior studies mainly focus on case retrieval, but the complete process of CBR should be given more attention. Framework research is still the mainstream, and there is a lack of applied research or case studies. The objective of this paper is to develop a CBR-based model, including case retrieval, reuse, revision and retention, for pre-control and decision-making related to construction safety accidents, and come up with novel methods for weight determination and similarity calculation to improve the effectiveness and veracity of case retrieval and case reuse. 

This paper is structured as follows: it starts with a brief introduction to the current research status and problems of employing CBR in construction safety management, followed by a literature review about safety hazard identification and CBR-based models. Then, the framework of a CBR model is proposed which elaborates the principles of CBR and implementation steps of case retrieval, reuse, revision and retention. Finally, the feasibility of the CBR model is tested with a real-world case, which demonstrates that the CBR technique is a highly promising tool for facilitating safety managers’ decision-making. 

## 2. Literature Review

### 2.1. Identification of Construction Safety Hazards

Identification of ubiquitous hazards plays a fundamental role in construction safety management [14]. Unsafe behavior of people and unsafe status of objectives are two direct factors that may lead to safety accidents. Previous researchers mainly focused on safety hazard categories and identification methods. Winge et al. indicated that the most identified causal factors are worker actions, risk management, immediate supervision, usability of materials or equipment, local hazards, worker capabilities, and project management [6]. Haslam et al. prioritized the factors contributing to construction accidents and found that workers or work teams could cause 70% of accidents followed by shortcomings of equipment (56%) and workplace issues (49%), while problems related to the suitability and condition of materials just accounted for 27% [5]. Williams et al. explored the causal factors of accidents from the perspective of stakeholders, and identified five factors: client-related, consultant-related, contractor-related, construction worker-related, and construction site-related [4]. Tariq S. Abdelhamid and John G. Everett identified the root cause of construction accidents, and attributed unsafe conditions to three causes: management actions, unsafe worker or coworker acts and non-human-related event(s) [15]. Patel and Jha determined 10 hazards, such as scaffolding and ladder usage, false work, roof work, with their corresponding physical attributes using the Delphi method [16]. Memon et al. investigated factors that influencing health and safety hazard in the construction industry, including personnel knowledge and professional skills, equipment related factors, operational procedures and organizational regulations [17]. In order to decrease people’s subjectivity in hazard identification, artificial intelligence has been introduced to facilitate the hazard identification process. Goh and Chua developed a CBR-based model, advocating the use of past knowledge in the form of past hazard identification and incident cases to improve the efficiency and quality of new hazard identification [18]. Kim et al. proposed an accident case retrieval system that can automatically generate queries based on the work, construction site conditions and laborers [19]. 

### 2.2. Case-Based Reasoning (CBR)

Case-based reasoning is a problem solving and learning method based on knowledge representation. It solves current problems by referring to the experience and knowledge of similar previous cases. As part of artificial intelligence theory, CBR is line with the trend of information development and has gained more attention from academic researchers [10]. 

There are limited studies which attempt to introduce CBR theory into construction safety management and risk management. Ying et al. developed a CBR model for safety risk management of subway operations, adopting a semantic network to describe potential risks from workers, physical systems and the environment to achieve case representation and retrieval [20]. Chen et al. proposed a framework of decision-support system for adjudicating fatal construction industry occupational accidents base on CBR method [21]. Virkki-Hatakka and Reniers developed a CBR-base platform software—Nextcase/safety—for taking measures to prevent safety accidents, and tested it successfully using a real accident case from the marine industry [22]. Goh and Chua aimed to identify the construction hazards using CBR, a framework including a knowledge representation scheme and an intelligent retrieval mechanism was developed where a linguistic structure is used to codify incident cases and past hazard identification, and similarity scoring is used to conduct case retrieval [23]. They also studied the adaptation and utilization process of CBR [18]. CBR was extended to risk management of subway projects, where the model aimed to identify risk categories and generate risk response strategies [24].

The key techniques of CBR generally include four aspects: case retrieval, case reuse, case revision and case retention [22]. In terms of case retrieval, Porter et al. constructed a network structure to describe the case [25]. Subsequently, Rodriguez and Vadera proposed a probabilistic exemple-based model by introducing Bayesian networks to develop a suitable representation and used probabilistic propagation for evaluating and retrieving exemples when a new case comes up [26]. Macedo and Cardoso adopted a causal connection arc to establish the adjacency matrix and drew the nested graph-structure model to represent the case [27]. The application of this approach in architectural design was further discussed. Similarity measures play a vital role in case retrieval, and Liao et al. focused on similarity measuring methods for CBR and proposed a hybrid similarity measure for comparing cases with a mixture of crisp and fuzzy features [28]. Ontology techniques enable one to define the structures of knowledge components and their relationship, which has been widely introduced in design cases for representing the problem universe of discourse [29]. Armaghanab suggested introducing the multi-criteria decision concept in problem representation description and proposed decision models such as the ELECTRE-I and ELECTRE-II based on knowledge acquisition, which could seek solutions from non-compensatory multi-criteria decision aids [30]. As for case reuse, Pérez et al. proposed a case-based reasoning scheme to extract and reuse design patterns by introducing a genetic algorithm which was used to optimize combinational logic circuits at the gate level [31]. Adeyanju et al. developed a Case Retrieval Reuse Net which could generate annotations to identify reusable text content that needed revision [32]. With regard to case revision, Jin et al. proposed a new adaptation method called adaptability-based FCA (AFCA) for solution feature values of retrieved cases by using decision tree technique and similarity values which were derived from a multi-algorithm-oriented hybrid SM strategy [33].

## 3. Model Development

**Figure 1:**
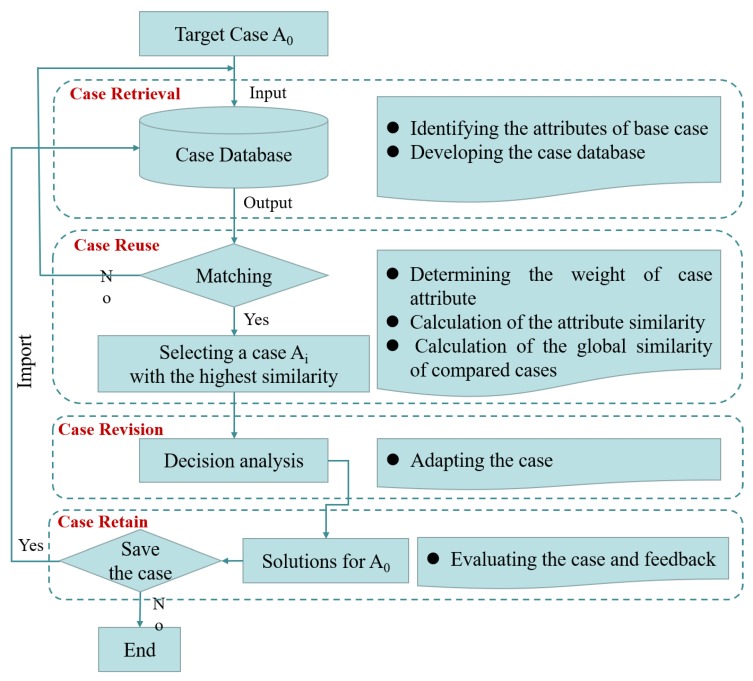
Logical framework and implementation step of the CBR model.

The accuracy of case retrieval determines the effectiveness of case reuse, and then determines the agility of beforehand decision-making about construction safety accidents. Hence, this paper concentrates on the implementations of case retrieval and case reuse, but also provides the principles of case revision and case retention. Figure 1 depicts the procedure for developing the proposed pre-control and decision-making model of construction safety accidents using CBR. The entire procedure can be divided into four phases, including seven detailed steps as described below.

Case Retrieval

Case retrieval aims to identify similar cases and aid decision-making for target cases by developing a case database. It requires all-round information with a modular structure in order to improve retrieval efficiency and facilitate case storage. The main task of case retrieval is to develop a case database by identifying ontology characteristics of both problem attributes and solution attributes. Chen et al. depicted the problem attributes using type of project, type of operation, cause of safety accident, number of casualties, and type of accident medium [21]. The solution attributes focus on the relationship between factors and strategy to deal with problems. Case retrieval is also regarded as a part of knowledge representation [23], hence, the methods which are widely used in knowledge representation, such as the memory network method, concept tree method, semantic network method [20] and framework system method can be recruited in case representation.

Case Reuse

Case reuse, also called case adaptation, intends to map the solution from previous cases to the target problem. It needs to be able to retrieve similar cases quickly to ensure model efficiency, and find a limited number of cases to ensure the effectiveness of the model. The main task of case reuse is to calculate the similarity between target cases and past cases by the following steps: determining the weight of case attributes, calculating the similarity of case attributes, and calculating the global similarity of compared cases.

Case Revision

Case revision means testing the new solution in the real world after having mapped previous solutions to the target situation. The similar previous cases need to be further scrutinized to determine whether they are suitable for the current situation and whether they can solve the target problem. If problem attribute of a similar case is consistent with the case to be analyzed, then the solution attributes of the similar case can support decision-making directly. If the similar case cannot fit the new background or scenario, the retrieved case needs to be adjusted.

Case Retention

After the solution has been successfully adapted to the target problem, it is necessary to store the resulting experience as a new case in the database. Case retention is a dynamic process of adding and removing cases aiming at improving the efficiency of the CBR model. As the number of past cases in the case database increases, similar and repeated cases occur. They not only take up space in the database, but also reduce the efficiency of case adaptation. Therefore, when the expense of case adaption outweighs the benefit it brings, it is suggested that the stored case in the database be deleted to decrease the redundancy.

### 3.1. Identifying the Attributes of Base Case

Safety is a state existing under a potential hazard condition, which is not absolutely stable and invariable. The stable state will be broken when the potential hazard state exceeds the limit of the endurable condition, at that time a hazardous state occurs and can cause safety accidents. This paper regards safety hazards as a main problem attribute of construction safety accident cases. How to describe the attributes of cases and map the pre-control measures against the accident causes are the key tasks in this section. Therefore, the hazard status set (HSS) and solution set (SS) of construction safety accidents are constructed, respectively.

#### 3.1.1. Establishment of Hazard Status set (HSS)

Safety hazards refers to the place, position, equipment or action where danger is likely to occur in the production process. The operation environment is an external potential hazard, which could be harmful to the physical condition of workers, such as occupational diseases. The machinery and equipment often pose threats to workers. The workers’ behaviors which violate safety regulations are also to blame.

According to the dynamic nature of hazard status, the safety hazards are divided into static hazards and dynamic hazards. Static hazards focus on the status of physical systems, such as the working site, equipment and materials. Dynamic hazards pay more attention on the status of workers and construction operation processes. We extracted 28 potential hazard indicators through an extensive prior literature search and allocated them into six clusters (shown in Table 1). 

The HSS is composed of hazard type and degree of severity. The degree of severity is used to describe the consequence of a safety accident, it needs to be quantified in the case presentation phase. The degree of severity of safety accident is denoted by the interval number (0–1), and the semantic expressions of hazard severity—“not serious”, “weakly serious”, “general”, “very serious”, “extremely serious” are quantized as (0–0.2], (0.2–0.4], (0.4–0.6], (0.6–0.8], (0.8–1], respectively.

#### 3.1.2. Establishment of Solution Set (SS)

**Figure 2:**
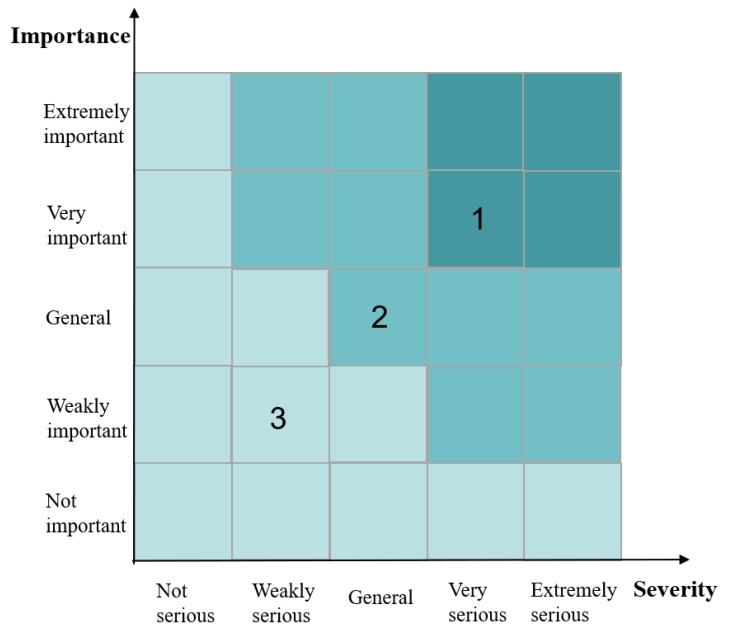
Evaluation principle of improvement strategies.

According to the different hazard status of safety accidents, the corresponding decision strategy is provided. Considering the countermeasures to hazard status are diverse for different cases, we generalize the solution attribute into three strategies: “General improvement”, “Critical improvement” and “Minor/no improvement” which are put forward according to the severity and importance of the hazard. The evaluation principles of improvement strategies are given in Figure 2. The X-axis represents the severity of a hazard status, which is divided into five levels from “not serious” to “extremely serious”, reflecting the harm of the accident consequences. The severity evaluation depends on peoples’ injuries or casualties, the impact on the safety and function of the engineering structure, the collapse or abandonment of engineering, the direct economic losses, the repair period and so on caused by safety accidents. The Y-axis represents the importance of a safety hazard. It is also known as the preference weight, as shown in Section 3.3, which is also measured by a five-scale. Hazard importance is contingent on the subjective judgment of experts based on the probability of a safety accident caused by a hazard and the influence degree of the safety hazard, such as a root cause with high weight value. It can be seen that the feasible zone is divided into three parts. For Zone 1 with high severity and high importance it is proposed that “Critical improvement” measures must be taken. For Zone 2 with at least one general severity or importance ranking, or weakly serious and highly important, or weakly important with high severity it is suggested to take “General improvement”, measures. For Zone 3 with low severity and importance we are supposed to apply “Minor/no improvement” measures. The SS is denoted as:

General improvement G = {A*_ij_*}
Critical improvement C = {A*_ij_*}
Minor/no improvement M = {A*_ij_*}

### 3.2. Developing the Case Database 

Case representation aims to codify past cases and identify safety hazard conditions [19]. This process is similar to that of knowledge representation [4]. Hence, a frame-based knowledge representation method is introduced to case database development. The advantages of this method are in representing structural knowledge, expressing special relationships between internal structural knowledge, and mapping all the related characters onto objects [47].

The frame is considered as a network with nodes and relations [48]. Slots and facets are two key elements in frame-based knowledge representation. Slots are used to represent the attributes of cases, and the function of facets is to indicate the value range and calculation method of slots [49]. A frame contains the information about how to use the frame, what to expect next and what to do if the expectation doesn’t achieve its objective. All information is contained in the slots or sides of the frame.

In term of this research context, the case is considered as the frame slot (indicated as *A_i_*), the hazard state of safety accident is set as the facet (indicated as *A_ij_*), and the facet value is evaluated by expert. According to the frame-based case representation method, six attribute slots of the hazard state and 28 attribute facets are given, the facet value is shown as *A_ij_*, where *i* = {1, 2, …, n}, n∈N. They are shown as follows:

Attribute slot *A*_1_ = {*A*_11_, *A*_12_, *A*_13,_ *A*_14_, *A*_15_}
Attribute slot *A_2_* = {*A*_21_, *A*_22_, *A*_23_}
Attribute slot *A_3_* = {*A*_31_, *A*_32_, *A*_33_}
Attribute slot *A*_4_ = {*A*_41_, *A*_42_, *A*_43_, *A*_44_, *A*_45_, *A*_46_, *A*_47_, *A*_48_, *A*_49_}
Attribute slot *A*_5_ = {*A*_51_, *A*_52_, *A*_53,_ *A*_54_, *A*_55_}
Attribute slot *A*_6_ = {*A*_61_, *A*_62_, *A*_63_}

### 3.3. Determining the Attribute Weight

**Figure 3:**
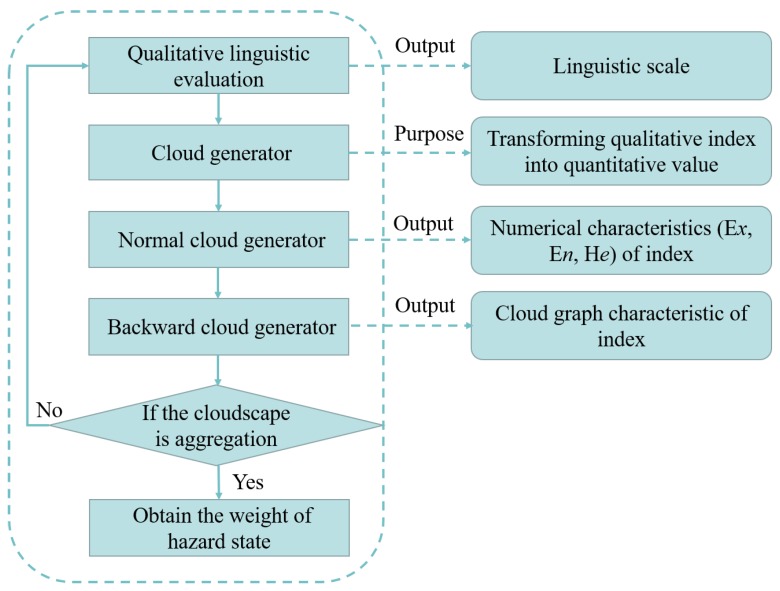
The steps of weight determination.

The usual method of weight determination such as in an analytic hierarchy process depends on limited information, and hardly takes the fuzziness and uncertainty of the evaluation object into consideration. However, the cloud model combines the fuzziness and randomness of evaluation objects effectively based on probability theory and fuzzy theory to realize the mapping between qualitative concepts and quantitative values [50], which can minimize the loss or distortion of information and improve the rationality of decision-making. Clouds are composed of many cloud droplets whose overall shape reflects the important characteristics of the qualitative concept [50,51]. Cloud droplets represent a quantitative description of a qualitative concept whose generation process intends to map the qualitative concept onto a quantitative value. The numerical characteristics of a cloud are usually demonstrated by the values of expectation (E*x*), entropy (E*n*), and hyper entropy (H*e*). E*x* represents the central value of a concept in the domain, so it is a fitting variable to reflect the value of a qualitative concept. E*n* accounts for the fuzziness, mirroring the range of values for acceptable qualitative concept. H*e* is used to measure the uncertainty of entropy, revealing the coherence of cloud drops in the domain. The procedures of weight determination of problem attribute /hazard state are shown in Figure 3.

#### 3.3.1. Constructing Linguistic Scale of Qualitative Indicators

Potential hazard indicators of construction safety accident are qualitative indexes which need expert evaluation according to a linguistic scale description. In terms of the importance of hazard indicators, a five-point scale with “Not important”,” Weakly important”,” General”,” Strongly important” and “Extremely important” linguistic characteristics is used. The degree of their values is quantified into [0,1], and the golden section method is employed to determine the range and cloud numerical characteristic of each linguistic scale [52] (shown in Table 2). 

#### 3.3.2. Quantitative Transformation of Expert Score 

This step will generate cloud droplet by normal cloud generator, and the quantitative position of droplet is represented by the degree of membership—a fuzzy concept.

Input: (*Ex, En, He, N*) 

N is the number of cloud droplets to generate.

Output: (Drop(x_1,_ C_T_(x_1_)), Drop(x_2,_ C_T_(x_2_),…, Drop(x_N,_ C_T_(x_N_)) 

(1) Generating a normal random number *En_j_^’^ = NORM (En, He)* with *En* as expected value and *He* as variance.

(2) Generating a normal random number *x_j_ = NORM (Ex, En_i_^’^)* with *Ex* as expected value and *En_i_^’^* as variance.

(3) Calculating degree of membership of drop x_i_:

(1) $$C_{T}\left(x_{i}\right)=\exp\left[-\frac{{\left(x_{i}-E_{x}\right)}^{2}}{2{\left(E_{n}^{\prime }{}_{{}_{i}}\right)}^{2}}\right]$$

(4) Repeating above steps until N cloud droplets are generated.

#### 3.3.3. Generating Numerical Characteristics

This step intents to generating numerical characteristics and cloud model by backward cloud generator.

Input: (Drop(x_1_, C_T_(x_1_)), Drop(x_2_, C_T_(x_2_),…, Drop(x_N_, C_T_(x_N_))

Output: (*Ex, En, He*)

(1) Generating expected value *Ex* by calculating the mean value *Ex* = Mean (x_i_):
  (2) $$E_{x}=\frac{1}{N}\sum_{i=1}^{N}x_{i}$$
(2) Generating entropy value *En* by calculating the variance *En* = STDEV (x_i_):
  (3) $$E_{n}=\sqrt{\frac{1}{N-1}{\sum_{i=1}^{N}\left(x_{i}-E_{x}\right)}^{2}}$$
(3) Generating hyper entropy by calculating the variance of *He* = STDEV(*En_i_^’^*):
  (4) $$E_{n_{i}}=\sqrt{\frac{-{\left(x_{i}-E_{x}\right)}^{2}}{2\ln\left(C_{T}\left(x_{i}\right)\right)}}$$
  (5) $$H_{e}=\sqrt{\frac{1}{N-1}\sum_{i=1}^{N}{\left(E_{n_{i}}-E_{n}\right)}^{2}}$$

#### 3.3.4. Forming Cloud Graph through Normal Cloud Generator

This step aims to form cloud graph under the principle of optimal numerical characteristics through normal cloud generator.

Input: *N* cloud droplets are created through selecting optimal numerical characteristics.

Output: Mapping the positions of *N* cloud droplets in the domain space, which is depicted by the membership C_T_(x_1_).

(6) $$E_{n_{i}}=NORM\left(E_{n},H_{e}{}^{2}\right)$$

(7) $$x_{i}=NORM\left(E_{n},E_{n_{i}}{}^{2}\right)$$

(8) $$\mu_{i}=\exp\left[-\frac{{\left(x-E_{x}\right)}^{2}}{2E_{n_{i}}{}^{2}}\right]$$

#### 3.3.5. Repeating the Above Processes until Generating *N* Droplets

The process needs to be repeated 28 times before the cloud model of all attribute facet weights is obtained. Ten industry experts (five safety team leaders, two project managers and three supervisors) with more than 5 years of safety management experience, were interviewed in order to conduct the weight determination process. Based on their knowledge and experience, the importance of hazard states was evaluated. Taking the weight determination process of attributes *A*_32_ “usability of materials” as an example, we got the evaluation results and numerical characteristics (*Ex, En, He*) shown in Table 3.

**Figure 4:**
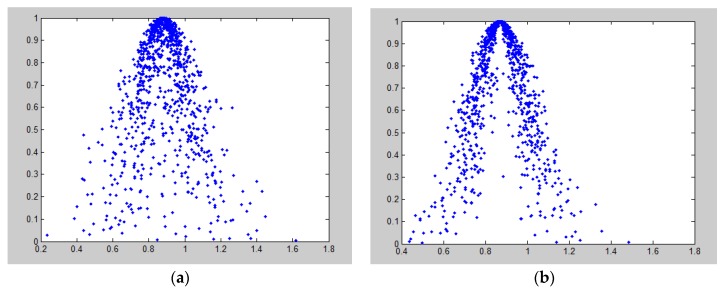
(**a**) The original cloud graph of attribute *A*_32_, (**b**) The updated cloud graph of attribute *A_32_.*

The membership degree of *x*_32_ towards the hazard severity is iteratively calculated by MATLAB following the above formulas. The cloud graph result is shown in Figure 4a. The droplet emerges the fog shape and with large divergence, which indicates that experts hold different views on the effect intension of “usability of materials”. Besides, the values of *En* and *He* are large which demonstrates that there is a significant randomness between cloud droplet position and the membership of linguistic scale. Due to the unreliable results, an extra round of expert interviews was conducted through e-mail and telephone. Finally, the numerical characteristics were adjusted to (0.870, 0.128, 0.040), showed in the updated Figure 4b. It is clear to see that the cloud graph generated by the normal cloud generator shows strong convergence. Ultimately, the weight of the attribute facet *A*_32_ is 0.87. 

Similarly, the weight of the other 27 attribute facets are obtained through repeating the above procedures (see Table 4).

### 3.4. Calculation of Attribute Similarity

Previous research has explored several approaches for similarity calculation, such as the taxonomy tree method [53], similarity scoring approach [21], and semantic network method [20]. However, most problem attributes and solution attributes in case presentation are text-based contents rather than numerical ones, and thus hard to quantify. In this research, the similarity scoring approach is introduced to calculate the similarity score between two hazard states based on a Chinese thesaurus. This approach employs a tree structure way to encode words that have the same or similar meaning [54]. The attribute similarity is calculated based on word sense similarities in a thesaurus [55]. Words and sentences are used as keywords for case retrieval instead of paragraph contents. Therefore, this paper provides a method for word level and sentence level similarity calculation.

#### 3.4.1. Word Level Similarity

The calculation formula of word similarity is shown as:

(9) $$SimW\left(w_{x},w_{y}\right)=\sum_{i=1}^{i}\delta_{i}\left(w_{x},w_{y}\right)\theta_{i}$$

$SimW\left(w_{x},w_{y}\right)$ represents the degree of similarity between two Chinese words; $\theta_{i}$ is the weight of words encoded in layer *i*; The value of $\delta_{i}\left(w_{x},w_{y}\right)$ depends on the encoded layer between words *w_x_* and *w_y_*:

(10) $$\delta_{i}\left(w_{x},w_{y}\right)=\{\begin{array}{c} 1,\\ 0, \end{array}\begin{array}{c} \;\mathrm{when}\;\mathrm{the}\;\mathrm{words}\;\mathrm{are}\;\mathrm{the}\;\mathrm{same}\;\mathrm{in}\;\mathrm{code}\;\mathrm{level}\;i\\ \;\mathrm{when}\;\mathrm{the}\;\mathrm{words}\;\mathrm{are}\;\mathrm{different}\;\mathrm{in}\;\mathrm{code}\;\mathrm{level}\;i \end{array}$$

The thesaurus contains roughly 70,000 Chinese words, covering three levels—large, medium, and small—with a tree structure. In order to facilitate case matching, the small level category are divided into different themes and synonyms.

#### 3.4.2. Sentence Level Similarity

By decomposing the structural components of Chinese sentences, the qualitative sentences can be converted into quantitative values. This research focuses on the content words in sentence because the description of hazard status mainly relies on content words. The quantitative expression of sentences is depicted as: S={N,V,A,M,Q,R}, the element represents “Sentences”, “Nouns”, “Verbs”, “Adjectives”, “Numerals”, “Quantifiers”, “Pronouns” successively.

First, the two sentences are expressed as $S_{1}=\left\{N_{1},V_{1},A_{1},M_{1},Q_{1},R_{1}\right\};$ $S_{2}=\left\{N_{2},V_{2},A_{2},M_{2},Q_{2},R_{2}\right\}$. Then, it is necessary to calculate the similarity of six elements in turn. This paper takes the calculation of nouns similarity between two sentences as an example. Supposing that sentence *s*_1_ and *s*_2_ has *m* and *n* nouns respectively, the set of nouns are denoted by $N_{1}=\left\{w_{11},w_{12},\ldots ,w_{1m}\right\};$ $N_{2}=\left\{w_{21},w_{22},\ldots ,w_{2n}\right\}.$ The similarity matrix of nouns is shown as follows:

(11) $$SimN\left(s_{1},s_{2}\right)=N_{1}\times N_{2}^{T}=\left[\begin{array}{ccc} w_{11}w_{12} & \cdots & w_{1m}w_{21}\\ \vdots & \ddots & \vdots \\ w_{11}w_{2n} & \cdots & w_{1m}w_{2n} \end{array}\right]$$

According to this characteristic matrix, the similarity of noun sets in sentences *s*_1_ and *s*_2_ can be formulated as follows, where *k* is the number of matrix elements:

(12) $$SimN\left(s_{1},s_{2}\right)=\frac{1}{k}\sum_{i=1}^{k}SimW\left(w_{1m},w_{2n}\right)$$

The similarities of other element in sentences, such as verbs, adjectives, etc., can also be obtained based on this rule. Finally, the whole sentence similarity between *s*_1_ and *s*_2_ is obtained by the weighted arithmetic mean method:

(13) $$SimS\left(s_{1},s_{2}\right)=\sum_{i=1}^{6}\beta_{i}SimS_{i}\left(s_{1},s_{2}\right)$$

$\beta_{i}$ is constant coefficient, which can be adjusted according to the different cases.

### 3.5. Calculation of Global Similarity 

Kolodner proposed the nearest neighbor method for case similarity calculations in 1993 [56]. This method is widely adopted in CBR. The calculation principle is as follows:

(14) $$Sim_{j}\left(C_{i},C_{j}\right)=\frac{\sum_{i=1}^{k}\omega \left(A_{i}\right)\times Sim_{i}\left(C_{i}\left(A_{i}\right),C_{j}\left(A_{i}\right)\right)}{\sum_{i=1}^{k}\omega \left(A_{i}\right)}$$

$\omega \left(A_{i}\right)$ is the weight of case attribute; $Sim_{i}\left(C_{i}\left(A_{i}\right),C_{j}\left(A_{i}\right)\right)$ is the similarity of attribute *A_i_* between a target case *C_i_* and past case *C_j_*.

### 3.6. Adapting the Case 

Due to complexity of the construction process and diversified latent hazards, though potential hazards could be identified as the same type in advance, the nature and severity of the safety accidents caused by them may be significantly different, so it is hard to find two coincident cases. In this situation, the retrieved similar case needs to be adapted for the context of the target problem.

According to different backgrounds of target cases, a threshold value $\mu_{0}$ is determined by referring to expert opinions, which is used to filter the similar cases [18]. If the similarity of the historical case is superior that of the target case ($\mu \rangle \mu_{0}$), then the calculated similarity is considered to be effective. However, if the similarity of the historical case is inferior that of the target case (i.e., $\mu \langle \mu_{0}$), then the calculated similarity is considered to be ineffective. When there are multiple past cases with high similarity, we should arrange them according to their similarity values, the closer the result is to 1 the more valuable the case for aiding decision making.

The method of case adaption is divided into three categories: solution addition, solution deletion, and both addition and deletion of solutions [57]. Solution addition means that there are too few measures to solve the current problem after case matching. It is necessary to increase the solutions according to the actual situation of the construction and expert opinions. Solution deletion refers to when there are too many solutions for pre-control of safety accidents to effectively solve the current problem. Some inessential solutions need to be deleted and an optimal strategy chosen according to expert opinions. Both addition and deletion of solution means that on the one hand, there are insufficient solutions for current problem solving, but on the other hand, the reference solution is not applicable to the target case. At this situation solution addition and deletion should be integrated.

### 3.7. Evaluation and Feedback

The CBR-based safety accident case database is an intelligent model, which can add and delete cases automatically to make case matching more accurate and efficient [21]. Case evaluation and feedback play a vital role in this mechanism. The incompleteness and redundancy of a case database are contradictory, but concepts are coordinated to some degree [58]. If the case database is deficient, it is highly possible we will find no case similar to the target case. On the contrary, if cases are added to the database without selection and evaluation, it will cause case duplication which wastes time during case retrieval and reduces the efficiency of case reuse. In fact, some specific type of cases should be given priority and be stored in the database rather than all feasible cases, which could prevent an exponential increase of the case database size and avoid database management difficulties of. It is noticeable that selecting and adding cases to the database poses a big challenge for the operational efficiency of the system.

## 4. Case Study 

The goal of the case study is to demonstrate how a most similar case can be retrieved based on the proposed knowledge representation and similarity calculation. A real-word case concerning a cultural square project is selected to conduct the case study. 

### 4.1. Description of Target Case

**Figure 5:**
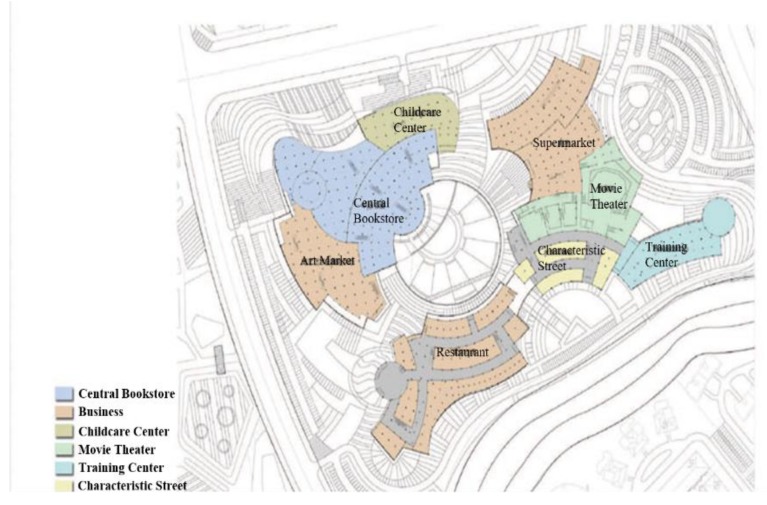
Layout and function of CSP.

The Cultural Square Project (CSP) is located in the northeast city of Harbin in China. It occupies 125,000 square meters of ground floor area and 155,000 square meters of total floor area. It is a complex project covering the functional areas of art market, characteristic street, cinema and supermarket (shown in Figure 5). The construction of this project faced great technical and management challenges due to the large scale of project, complex building structure, high aesthetic and appearance requirements, superior quality of decorative materials, multiple participants and a complicated organizational relationship.

The target case is articulated. The roof of the 4th floor patio was originally designed as a sightseeing well, before the owner proposed to change it into a concrete slab. During the construction process the safety manager found that the scaffolding workers had installed a scaffold as support system using the conventional method which might result in a large distance between supporting bars. Therefore, the target case is set as “the distance of vertical supporting bars exceeds the requirement of the construction plans”, and we input this sentence into the CBR-based model to conduct case retrieval and adaptation.

### 4.2. Research Finding and Discussion

In order to simplify the implementation process of CBR-based decision model, this study assumes that there are just two past cases *C*_1_ and *C*_2_ in the case database related to hazard *A*_51_ “Maturity of construction technique” and *A*_54_ “Mold installation and usage” (show in Table 5).

Supposing the input sentence of target case is represented as *s*_0_, *s*_0_ = “The distance of the vertical supporting bars exceeds the requirement of the construction plan”. The matching sentence in previous cases *C*_1_ and *C*_2_ are denoted as *s*_1_ and *s*_2_, *s*_1_ = “The quality of the construction formwork disobeys the requirement of the construction plan”, *s*_2_ = “The distance of vertical formwork exceeds the requirement of the construction plan”.

Step1: Similarity calculation of nouns

The noun set is represented as:

*N*_0_ = {formwork, vertical bar, distance, plan, requirement}
*N*_1_ = {formwork, quality, plan, requirement}
*N*_2_ = {formwork, vertical bar, space, plan, requirement}

The nouns are encoded as “Formwork” B/o/15/01/05/01, “Vertical bar” B/b/04/03/05/03, “Quality” D/d/12/01/01/01, “Distance” D/n/02/01/01/01, “Space” D/n/02/01/01/02, “Plan” D/k/17/02/17/01, “Requirement” D/f/07/02/05/03. According to equations (9) and (10), the similarity of nouns between *N*_0_ and *N*_1_ is shown in Table 6, and the similarities of nouns between *N*_0_ and *N*_2_ are shown in Table 7.

According to the formulation (12), the noun’s similarities of *N*_0_ and *N*_1_ are:

$SimN\left(s_{0},s_{1}\right)=\frac{1}{20}\sum_{i=1}^{20}SimN\left(w_{0i},w_{1j}\right)=0.17;$

$SimN\left(s_{0},s_{2}\right)=\frac{1}{25}\sum_{i=1}^{25}SimN\left(w_{0i},w_{2j}\right)=0.25$

Step 2: Similarity calculation of verbs:

The set of verbs is denoted as *V*_0_ = {exceed}, *V*_1_ = {disobey}, *V*_2_ = {exceed}.

The verbs are encoded as “exceed” J/b/04/01/01/01, “disobey” J/c/03/01/01/02.

The similarity of verbs between *V*_0_ and *V*_1_ is Sim (exceed, disobey) = 1/6

The similarity of verbs between *V*_0_ and *V*_2_ is Sim (exceed, exceed) = 1

The similarity of verb set is $SimV\left(s_{0},s_{1}\right)=0.17;$ $SimV\left(s_{0},s_{2}\right)=1$

Supposing that $\beta_{N}=0.75$, $\beta_{V}=0.78$, The similarity of sentence between *s_0_* and *s*_1_ is,

$SimS\left(s_{0},s_{1}\right)=\sum_{i=1}^{2}\beta_{i}SimS_{i}\left(s_{0},s_{1}\right)=0.75\times 0.17+0.78\times 0.17=0.26$

The similarity of sentence between S*_0_* and S*_1_* is, 

$SimS\left(s_{0},s_{2}\right)=\sum_{i=1}^{2}\beta_{i}SimS_{i}\left(s_{0},s_{2}\right)=0.75\times 0.25+0.78\times 1=0.97$ The results $Sim\left(s_{0},s_{2}\right)\;\rangle \;Sim\left(s_{0},s_{1}\right)$ indicated that the similarity between the case *C*_0_ and *C*_2_ is greater than that between the case *C*_0_ and *C*_1_, so the historical case *C*_2_ is the most similar case. 

The solution scheme of *C*_2_ includes suspending construction, improving the construction plan approval system, intensifying the supervision and inspection. The case retrieval and reuse are effective after expert re-evaluation. According to the solution measures obtained from *C*_2,_ suitability evaluation should be further carried out for ensuring the solution strategies are supported for hazard prevention of the target case. 

(1) Construction suspension

Referring to the relevant construction safety management regulation, when in a project there exists a potential hazard which could cause a fatal safety accident or pose great threat to life or property without construction suspension for rectification [59], under the confirmation of an engineer, the project needs to be suspended until the safety hazard has been eliminated. This strategy is fit for the CSP case, which suggests that engineers and technical workers should conduct safety inspections, and demonstrations for the formwork installation plans. 

(2) Improvement of the approval system for the construction plans

This measure aims to establish the safety responsibility guarantee system to clear the main responsibility among stakeholders. Particularly, the new construction plan cannot be implemented until the engineer has approved it. This solution strategy is also suitable for the target case. Due to temporary change of design plan by the owner, re-examination and approval of a new formwork plan is required rather than carrying out the original plan.

(3) Strengthening the supervision and inspection

Safety managers or supervisors play an essential role in the identification and pre-control of potential hazards [38]. Besides, they can rectify the unsafe behavior of construction workers. Hence, this measure could also match with the target case.

(4) Supplementary measures related to human factors

Additionally, some extra alternatives are put forward in the context of CSP. The root causes of potential hazards cannot be separated from human factors [15]. With regard to the target case, the formwork installation plan and usage needs to be updated after design changes, and under this circumstance, workers need receive technical clarification and new safety training. Therefore, the hazards related to workers should also been given attention, such as *A*_44_, *A*_45_, *A*_46_.

In conclusion, the pre-control measures that in the target case could be taken mainly include construction suspension for improvement, upgrading the approval system for the construction plan, strengthening supervision and inspection, improving safety consciousness of workers, intensifying safety training and technical guidance for workers.

## 5. Conclusions 

Though prevention of safety accidents plays an important role in construction safety management, few studies have developed a system framework of a model to facilitate construction safety practitioners to make pre-decision making. This study focuses on the mechanism of safety accidents and develops a pre-decision-making framework by applying the CBR method. According to the principles of CBR, the case retrieval and case reuse are the main research tasks. First of all, the case representation structure was formed. A base case is identified from the hazards state attribute and solution attribute. Using the frame-based knowledge representation method, the hazards state is divided into six frame slots covering static and dynamic hazards, and then it was subdivided into 28 attribute facets. According to the attribute values of different attributes in each case, we can provide targeted solutions for different situations in different cases. Besides, a case reuse system is developed. The cloud model is used to determine the weight of hazard attributes. The normal cloud generator and backward cloud generator are used to convert qualitative concepts and qualitative values. A MATLAB program is used to calculate the weight of hazards and analyze their numerical characteristics. Finally, a CSP in Harbin was simulated according to the proposed implementation process of the CBR-based decision-making model. The most similar case which is related to scaffolding deviation was retrieved and reused.

By implementing CBR, a conceptual framework for a safety accident pre-decision making system was developed. The developed framework is expected to provide a platform for construction practitioners to understand the hazards of safety accidents systematically and also offers valuable information for safety management. The proposed framework also serves as a reference for possible safety accidents in new cases based on the experience obtained from previous cases. 

Three limitations in this research should be noted. First, this research mainly focuses on the processes of case retrieval and case reuse and the other two processes, case revision and case retention are not explained in details. Besides, in terms of case study, the case database is based on an assumption, and the number of previous cases is small, which weakens the reliability of the proposed CBR-based model. Moreover, the case study in this research just simulated the case reuse process, while other processes need to be further verified.

Future research should expand the CBR-based decision-making framework and develop a complete model covering case retrieval, case reuse, case revision and case retention. It is worth exploring case addition and deletion in order to improve the efficiency of case adaptation. Additionally, an operational system should be further developed for practical applications. More complicated target cases should be selected to verify the feasibility of the CBR-base decision-making model for safety accident prevention.

## Figures and Tables

**Table 1:** Potential hazard indicators of construction safety accidents.

Dimension	Indicator	Sub-Indicator	Source
Static Hazard	Workplace *A*_1_	Abnormal hydrogeology condition *A*_11_	[34]
		Abnormal weather conditions *A*_12_	[6]
		Site conditions (including electricity, lighting, ventilation and sanitation) *A*_13_	[5]
		Site layout and space *A*_14_	[5]
		Housekeeping *A*_15_	[4]
	Equipment *A*_2_	Condition of equipment *A*_21_	[5,35]
		Usability of equipment *A*_22_	[4,5]
		Suitability of equipment *A*_23_	[4,5]
	Materials *A*_3_	Condition of materials *A*_31_	[4,5]
		Usability of materials *A*_32_	[4,5]
		Suitability of materials *A*_33_	[4,5]
Dynamic Hazard	Worker’s unsafe behavior *A*_4_	Knowledge and skills *A*_41_	[4,6]
		Physical health or fatigue *A*_42_	[5]
		Mental health *A*_43_	[4]
		Safety consciousness or awareness *A*_44_	[36]
		Safety training for workers *A*_45_	[15,35,37]
		Technical guidance for workers *A*_46_	[4,38]
		Education of workers *A*_47_	[4,37]
		Legal consciousness *A*_48_	[37]
		Improper supervision *A*_49_	[5,39]
	Construction scheduling *A*_5_	Maturity of construction technique *A*_51_	[34]
		Stability of working platform *A*_52_	[38]
		Compressed construction schedule *A*_53_	[40]
		Mold installation and usage *A*_54_	[41]
		Scaffolding installation and usage *A*_55_	[42,43]
	Operation management *A*_6_	Operation of lifting equipment *A*_61_	[44]
		Operation of processing machinery *A*_62_	[45]
		Operation of measuring apparatus *A*_63_	[46]

**Table 2:** Linguistic scale of hazard state.

Importance Rating	Not Important	Weakly Important	General	Strongly Important	Extremely Important
Scope of weight coefficient	0.0–0.2	0.2–0.4	0.4–0.6	0.6–0.8	0.8–1.0
*Ex*	0.000	0.309	0.500	0.691	1.000
*En*	0.1031	0.0640	0.0390	0.0640	0.1031
*He*	0.013	0.008	0.005	0.008	0.013

**Table 3:** Numerical characteristics of attributes facet *A*_32_.

Attribute Facet	Exp.1	Exp.2	Exp.3	Exp.4	Exp.5	Exp.6	Exp.7	Exp.8	Exp.9	Exp.10
*A*_32_	0.8	0.6	0.8	1.0	0.8	1.0	1.0	0.8	1.0	1.0
*Ex*	0.88									
*En*	0.14									
*He*	(1.000, 0.1031, 0.013)									

**Table 4:** Weight of attribute facets.

Attribute Slot	Attribute Facet	Weight
Workplace *A*_1_	Abnormal hydrogeology condition *A*_11_	0.84
	Abnormal weather conditions *A*_12_	0.86
	Site conditions (including electricity, lighting, ventilation and sanitation) *A*_13_	0.86
	Site layout and space *A*_14_	0.75
	Housekeeping *A*_15_	0.65
Equipment *A*_2_	Condition of equipment *A*_21_	0.79
	Usability of equipment *A*_22_	0.88
	Suitability of equipment *A*_23_	0.68
Materials *A*_3_	Condition of materials *A*_31_	0.87
	Usability of materials *A*_32_	0.88
	Suitability of materials *A*_33_	0.75
Worker’s unsafe behavior *A*_4_	Knowledge and skills *A*_41_	0.78
	Physical health or fatigue *A*_42_	0.76
	Mental health *A*_43_	0.54
	Safety consciousness or awareness *A*_44_	0.94
	Safety training for workers *A*_45_	0.89
	Technical guidance for workers *A*_46_	0.77
	Education of workers *A*_47_	0.48
	Legal consciousness *A*_48_	0.56
	Improper supervision *A*_49_	0.82
Construction scheduling *A*_5_	Maturity of construction technique *A*_51_	0.80
	Stability of working platform *A*_52_	0.85
	Compressed construction schedule *A*_53_	0.73
	Mold installation and usage *A*_54_	0.87
	Scaffolding installation and usage *A*_55_	0.91
Operation management *A*_6_	Operation of lifting equipment *A*_61_	0.84
	Operation of processing machinery *A*_62_	0.52
	Operation of measuring apparatus *A*_63_	0.44

**Table 5:** Descriptions of previous *C*_1_ and *C*_2_.

Previous Case	*C*_1_	*C*_2_
Hazard state	The quality of the construction formwork does not meet the construction plan requirement	The space of vertical bars in the formwork exceeds the requirements of the construction plan
Accident	Collapse of the formwork system	Collapse of the formwork system
Consequence	Eight persons died, three persons were injured, resulting in a direct economic loss of 3.394 million Yuan	Four persons died, five persons were injured, resulting in a direct economic loss of 1.50 million Yuan
Solution	Critical improvement (suspend construction, control materials strictly, scrutinize the construction plan strictly, strength safety management)	Critical improvement (suspend construction, improve the approve system of construction plan, intensify the supervision and inspection)

**Table 6:** Similarity of nouns between *N*_0_ and *N_1._*

Sim	Formwork	Vertical Bar	Distance	Requirement	Quality	Scheme
formwork	1	1/6	0	0	0	1
vertical bar	0	0	1/6	1/6	1/6	0
distance	0	0	1/6	1	1/6	0
requirement	0	0	1/6	1/6	1	0
quality	1	1/6	0	0	0	1
plan	0	0	1/6	1/6	1/6	0

**Table 7:** Similarity of nouns between *N*_0_ and *N_1._*

Sim	Formwork	Vertical Bar	Distance	Requirement	Space	Scheme
formwork	1	1/6	0	0	0	1
vertical bar	1/6	1	0	0	0	1/6
distance	0	0	5/6	1/6	1/6	0
requirement	0	0	1/6	1	1/6	0
space	0	0	1/6	1/6	1	0
plan	1	1/6	0	0	0	1
